# The Open Window

**Published:** 1913-08

**Authors:** 


### The Open Window

Dr. W. W. Roach, Medical Inspector in the Philadelphia Public Schools, with the co-operation of teachers and parents, recently made an experiment to determine the value of cold fresh air in school rooms, which was reported in the *American Journal of Public Health.* He opened the windows at top and bottom, and kept them open throughout the winter. The room was shut off from the heating plant of the building except on the occasional days when the temperature fell below forty-five degrees; but children wore extra wraps and had frequent drills and exercises.

In another room, the pupils were of the same grade and of about the same number, but the room was heated and ventilated according to the usual methods. The pupils in both rooms were normally healthy children from the same kind of homes, so that the test was as fair as possible.

From September 30 to December 20, 1912, the pupils in the open window room gained in weight on an average about two pounds, those in the warm air room a trifle less than one pound. The pupils in the open room kept wholly free from colds and were much more regular in attendance than the others. They were also more alert, free from day-dreaming, quicker to learn, needed less review work and were better behaved. In health and happiness, in development both of mind and body, the children of the room with open windows had a clear advantage over the others.

As a result the School Board has authorized the establishment of open window classes in several other Philadelphia schools.

(Note—In commending this observation, which corroborates similar experiments in Chicago and elsewhere, we wish to add a caution against too literal imitations in cold climates. It is well to observe carefully the limit of 45°F., and it is possible that good ventilation with higher temperature might be found even more desirable.)

